# A Partitioned Finite Difference Method for Heat Transfer with Moving Line and Plane Heat Sources

**DOI:** 10.3390/e28020179

**Published:** 2026-02-04

**Authors:** Jun Li, Yingjun Jiang

**Affiliations:** School of Mathematics and Statistics, Changsha University of Science and Technology, Changsha 410114, China; jiangyingjun@csust.edu.cn

**Keywords:** moving heat source, finite difference method, jump condition, heat conduction

## Abstract

This study proposes an efficient numerical scheme for simulating heat transfer governed by the diffusion equation with moving singular sources. The work addresses two-dimensional problems with line sources and three-dimensional problems with plane sources, which are prevalent in irreversible thermodynamic processes. Developed within a finite difference framework, the method employs a partitioned discretization strategy to accurately resolve the solution singularity near the heat source—a region critical for precise local entropy production analysis. In the immediate vicinity of the source, we analytically derive and incorporate the solution’s “jump” conditions to construct specialized finite difference approximations. Away from the source, standard second-order-accurate schemes are applied. This hybrid approach yields a globally second-order convergent spatial discretization. The resulting sparse system is efficient for large-scale simulation of dissipative systems. The accuracy and efficacy of the proposed method are demonstrated through numerical examples, providing a reliable tool for the detailed study of energy distribution in non-equilibrium thermal processes.

## 1. Introduction

In recent decades, the widespread adoption of automation in industrial processing and advanced manufacturing has spurred significant research interest in heat transfer under concentrated energy inputs. In particular, thermal modeling involving moving heat sources is critical for irreversible processes such as welding, laser cladding, metal cutting, and additive manufacturing [1,2,3]. Furthermore, applications have expanded into cutting-edge fields including photolithography, laser medicine, and biomedical therapies [4,5,6,7,8], underscoring the broad relevance of this fundamental problem in energy and information transfer systems.

The study of heat conduction due to moving sources dates back to the early 20th century. Rosenthal’s seminal work provided linear analytical solutions for point, line, and surface sources moving at constant velocity in semi-infinite domains [9]. This foundation has been extended by numerous studies: Jaeger established classical steady-state models for moving band and planar sources [10], a framework later systematized by Hou for various geometries and intensity distributions under transient and steady-state conditions [11]. Analytical solutions for three-dimensional transient fields have been derived for finite-thickness plates [12,13] and for various source types (e.g., point, ellipsoidal) using Green’s function methods [14]. To address the limitations of semi-infinite domain assumptions, analytical tools incorporating boundary effects have also been developed [15]. Other notable contributions include closed-form solutions for orthotropic solids [16] and a unified framework based on generalized incomplete gamma functions [17].

Despite their theoretical value, analytical methods are often constrained by their dependence on specific Green’s functions and involve complex integral operations, limiting their practicality for complex geometries or source motions. Consequently, developing robust and efficient numerical methods has become an essential pathway for analyzing the evolving energy states and dissipation in these systems.

Significant progress has been made in numerical simulations for moving point (concentrated) heat sources. Recent advancements include an overlapping grid-based element-free Galerkin method for precise temperature field capture near the source [18], and a hybrid element-free Galerkin–finite element method that enhances computational efficiency for three-dimensional nonlinear problems [19]. The finite element method has been used to simulate thermal stress in selective laser melting [20], and the extended finite element method has been applied to analyze crack-tip evolution during welding [21]. Meshless methods, such as the element-free Galerkin method, have successfully simulated temperature fields in welding processes [22,23,24]. Additionally, a moving mesh method has been developed to efficiently handle point sources following complex trajectories [25].

For steady-state problems with line sources, boundary element methods [26,27,28,29,30] and fundamental solution methods [31,32,33] have been employed effectively. However, a notable gap remains in the systematic development of numerical solvers for heat transfer problems involving moving line (2D) and moving plane (3D) heat sources. This gap limits our ability to perform detailed, high-fidelity simulations of entropy generation and energy flow in such continuous-source systems, motivating the present work.

In this paper, we introduce a novel finite difference method designed specifically for two- and three-dimensional heat transfer with moving line and plane sources. Our core innovation is a partitioned discretization strategy that treats regions near and far from the heat source differently. Adjacent to the source, we analyze the solution’s ”jump” properties—linked to local discontinuities in heat flux—to construct tailored first-order difference approximations. In distant regions, standard second-order central differences are applied. This strategy achieves global second-order spatial convergence. The resulting discrete system features a sparse matrix structure, amenable to efficient solvers like multigrid methods, and is readily extensible to more complex scenarios involving convection or nonlinearities. The method provides an efficient computational tool to explore the thermodynamic and informational aspects of heat diffusion from moving singularities.

This paper considers the following heat conduction equation with a moving singular source,

(1) $$u_{t}-\Delta u=\delta_{L_{t}}\left(p\right)q\left(p,t\right),\;p\in \Omega ,\;0<t\leq T,$$

subject to the following initial and boundary conditions:

(2) $$u\left(p,0\right)=u_{0},\;p\in \Omega ,$$

(3) $${u|}_{\partial \Omega }=0,$$

where Ω denotes a domain in $R^{n}$ (n=2,3), ∂Ω denotes the boundary of Ω, and $u_{0}$ is a known function. $L_{t}$ represents the heat source line (for n=2) or heat source plane (for n=3) at time *t*; $\delta_{L_{t}}$ represents a two- or three-dimensional Dirac delta function. It is infinite on $L_{t}$, zero in the region $\Omega \setminus L_{t}$, and its integral along a normal line segment that intersects $L_{t}$ at exactly one point equals 1. The function *q* denotes the heat source intensity.

The rest of this paper is organized as follows: In Section 2, we illustrate our method through constructing the finite difference discretization scheme for two-dimensional moving straight-line heat source problems; in Section 3, we validate the convergence accuracy of the proposed method through numerical examples, including both two-dimensional moving line and three-dimensional moving plane heat source problems; in Section 4, we summarize the work and provide an outlook for future research.

## 2. Construction of the Finite Difference Scheme

For conciseness, this section elaborates the construction process of the finite difference discretization scheme for solving the two-dimensional heat conduction problem with a moving straight-line heat source. The method can be directly generalized to general two-dimensional curve heat source and three-dimensional surface heat source problems. Its effectiveness will be validated in Section 3 through various numerical examples, including two-dimensional moving line and three-dimensional moving plane heat sources.

Equation (1) with a moving straight-line heat source in two-dimensional space can be written as

(4) $$u_{t}-\Delta u=\delta_{L_{t}}\left(x,y\right)q\left(x,y,t\right),\;\left(x,y\right)\in \Omega ,\;0<t\leq T,$$

subject to the following initial and boundary conditions:

(5) $$u\left(x,y,0\right)=u_{0},\;\left(x,y\right)\in \Omega ,$$

(6) $${u|}_{\partial \Omega }=0,$$

where Ω=[a,b]×[c,d], with a,b,c,d being constants; $L_{t}$ represents the moving line given by

(7) xcosα+ycosβ+Vt+D=0,

$\vec{n}=\left(\cos\alpha ,\cos\beta \right)$ is a unit normal vector of $L_{t}$; V≥0 and *D* are constants which, together with other coefficients, determine the line’s velocity; V=0 and V≠0 correspond to steady-state and transient heat sources, respectively.

**Figure 1:**
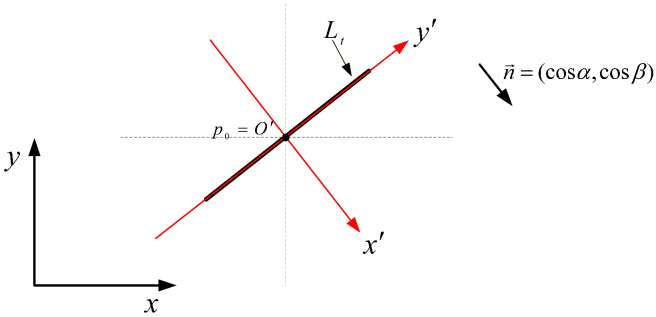
New coordinate system $x^{\prime }O^{\prime }y^{\prime }$.

For an arbitrary point $p_{0}\left(x_{0},y_{0}\right)$ on the line $L_{t}$, a new Cartesian coordinate system $x^{\prime }O^{\prime }y^{\prime }$ (see, Figure 1) is established with $p_{0}$ as the origin $O^{\prime }$, such that $\vec{n}$ is the unit vector aligned with the $x^{\prime }$-axis, and (−cosβ,cosα) is the unit vector aligned with the $y^{\prime }$-axis. It is clear that rotating the $x^{\prime }$-axis $90^{\circ }$ counterclockwise about $O^{\prime }$ yields the $y^{\prime }$-axis. The coordinates of $p_{0}$ in the $x^{\prime }O^{\prime }y^{\prime }$ system are (0,0). The coordinate mapping between the xOy and $x^{\prime }Oy^{\prime }$ systems is

(8) $$\left\{\begin{array}{c} x\left(x^{\prime },y^{\prime }\right)=x_{0}+x^{\prime }\cos\alpha -y^{\prime }\cos\beta ,\\ y\left(x^{\prime },y^{\prime }\right)=y_{0}+x^{\prime }\cos\beta +y^{\prime }\cos\alpha . \end{array}\right.$$

In the new coordinate system, Equation (4) becomes

(9) $$u_{t}-u_{x^{\prime }x^{\prime }}-u_{y^{\prime }y^{\prime }}=\delta_{L_{t}}\left(x\left(x^{\prime },y^{\prime }\right),y\left(x^{\prime },y^{\prime }\right)\right)q\left(x\left(x^{\prime },y^{\prime }\right),y\left(x^{\prime },y^{\prime }\right),t\right),$$

where $\delta_{L_{t}}\left(x\left(x^{\prime },0\right),y\left(x^{\prime },0\right)\right)=\delta \left(x^{\prime }\right)$, with δ being the one-dimensional Dirac delta function.

Equation (7) for the moving line $L_{t}$ can also be viewed as a plane equation in three-dimensional space (treating *t* as a variable). Denote this plane as Π, with a normal vector $\vec{m}=\left(\cos\alpha ,\cos\beta ,V\right)$. The plane Π divides the computational domain Ω×(0,T] into two regions:

$$\Omega^{+}=\left\{\left(x,y,t\right)\in \Omega \times \left(0,T\right];x\cos\alpha +y\cos\beta +Vt+D>0\right\}$$

and

$$\Omega^{-}=\left\{\left(x,y,t\right)\in \Omega \times \left(0,T\right];x\cos\alpha +y\cos\beta +Vt+D<0\right\}.$$

Based on the properties of solutions to heat conduction problems, we can assume that the solution *u* is sufficiently smooth within both $\Omega^{+}$ and $\Omega^{-}$, and that *u* is a sufficiently smooth bivariate function on the plane Π. However, the directional derivative of *u* normal to the plane Π is discontinuous near $L_{t}$. To describe this discontinuity, we introduce the concept of the “jump” of a function value (see ref. [34]). Assume a function g(x,y,t) is continuous on $\Omega^{+}$ and $\Omega^{-}$. If a moving point (x,y,t) passes from region $\Omega^{-}$ through point $P_{0}\left(x_{0},y_{0},t_{0}\right)$ on plane Π into region $\Omega^{+}$, the jump of g(x,y,t) at $P_{0}$, denoted ${\left[g\right]}_{P_{0}}$, is defined as

(10) $${\left[g\right]}_{\left(x_{0},y_{0},t_{0}\right)}=\lim_{\varepsilon \to 0^{+}}\left(g\left(P_{0}+\varepsilon \vec{m}\right)-g\left(P_{0}-\varepsilon \vec{m}\right)\right).$$

The point $p_{0}\left(x_{0},y_{0}\right)$ can be considered as a point on the line $L_{t_{0}}$. Regarding point $p_{0}\left(x_{0},y_{0}\right)$, establish the new coordinate system $x^{\prime }O^{\prime }y^{\prime }$ as described above. Setting $y^{\prime }=0$, $t=t_{0}$ in Equation (9), and integrating both sides with respect to $x^{\prime }$ over the interval (−ϵ,ϵ) for any ϵ>0, we obtain

(11) $$\begin{array}{cc} & \int_{-\epsilon }^{\epsilon }\left(u_{t}\left(x\left(x^{\prime },0\right),y\left(x^{\prime },0\right),t_{0}\right)-u_{x^{\prime }x^{\prime }}\left(x\left(x^{\prime },0\right),y\left(x^{\prime },0\right),t_{0}\right)-u_{y^{\prime }y^{\prime }}\left(x\left(x^{\prime },0\right),y\left(x^{\prime },0\right),t_{0}\right)\right)dx^{\prime }\\ = & \int_{-\epsilon }^{\epsilon }\delta_{L_{t_{0}}}\left(x\left(x^{\prime },0\right),y\left(x^{\prime },0\right)\right)q\left(x\left(x^{\prime },0\right),y\left(x^{\prime },0\right),t_{0}\right)dx^{\prime }\\ = & \int_{-\epsilon }^{\epsilon }\delta \left(x^{\prime }\right)q\left(x\left(x^{\prime },0\right),y\left(x^{\prime },0\right),t_{0}\right)dx^{\prime }\\ = & q\left(x\left(0,0\right),y\left(0,0\right),t_{0}\right)=q\left(x_{0},y_{0},t_{0}\right). \end{array}$$

Since $u_{t}$ is bounded within $\Omega^{+}$ and $\Omega^{-}$, we have

(12) $$\int_{-\epsilon }^{\epsilon }u_{t}\left(x\left(x^{\prime },0\right),y\left(x^{\prime },0\right),t_{0}\right)dx^{\prime }\to 0\;\left(\epsilon \to 0^{+}\right).$$

Since the $y^{\prime }$-axis is parallel to the line $L_{t}$, $u_{y^{\prime }y^{\prime }}$ is continuous at point $P_{0}$, yielding

(13) $$\int_{-\epsilon }^{\epsilon }u_{y^{\prime }y^{\prime }}\left(x\left(x^{\prime },0\right),y\left(x^{\prime },0\right),t_{0}\right)dx^{\prime }\to 0\;\left(\epsilon \to 0^{+}\right).$$

Direct integration gives

(14) $$\int_{-\epsilon }^{\epsilon }u_{x^{\prime }x^{\prime }}\left(x\left(x^{\prime },0\right),y\left(x^{\prime },0\right),t_{0}\right)dx^{\prime }=u_{x^{\prime }}\left(x\left(\epsilon ,0\right),y\left(\epsilon ,0\right),t_{0}\right)-u_{x^{\prime }}\left(x\left(-\epsilon ,0\right),y\left(-\epsilon ,0\right),t_{0}\right).$$

Combining (10), (11), (12), (13), and (14), and considering the limit $\varepsilon \to 0^{+}$, we obtain

(15) $${\left[u_{x^{\prime }}\right]}_{\left(x_{0},y_{0},t_{0}\right)}=-q\left(x_{0},y_{0},t_{0}\right).$$

Directly from solution properties, we have

(16) $${\left[u_{y^{\prime }}\right]}_{\left(x_{0},y_{0},t_{0}\right)}=0,\;{\left[u_{y^{\prime }y^{\prime }}\right]}_{\left(x_{0},y_{0},t_{0}\right)}=0.$$

From Equation (9) it follows that

(17) $${\left[u_{t}\right]}_{\left(x_{0},y_{0},t_{0}\right)}={\left[u_{x^{\prime }x^{\prime }}\right]}_{\left(x_{0},y_{0},t_{0}\right)}.$$

Combining the coordinate transformation (8) with (15), we get

(18) $${\left[u_{x}\right]}_{\left(x_{0},y_{0},t_{0}\right)}={\left[u_{x^{\prime }}\right]}_{\left(x_{0},y_{0},t_{0}\right)}\cos\alpha =-q\left(x_{0},y_{0},t_{0}\right)\cos\alpha$$

and

(19) $${\left[u_{y}\right]}_{\left(x_{0},y_{0},t_{0}\right)}={\left[u_{x^{\prime }}\right]}_{\left(x_{0},y_{0},t_{0}\right)}\cos\beta =-q\left(x_{0},y_{0},t_{0}\right)\cos\beta .$$

From the properties of the heat conduction equation solution, for any $(x_{0},y_{0},t_{0})\in \Pi$ and any vector $\vec{l}$ perpendicular to the normal vector $\vec{m}$, we have

(20) $${\left[\frac{\partial u}{\partial \vec{l}}\right]}_{\left(x_{0},y_{0},t_{0}\right)}=0.$$

Clearly, (−cosβ,cosα,0) is a vector perpendicular to $\vec{m}$. Indeed, when $\vec{l}=\left(-\cos\beta ,\cos\alpha ,0\right)$, (20) gives the first equation in (16). Via the cross product, another vector perpendicular to $\vec{m}$ is obtained:

$$\vec{m}\times \left(-\cos\beta ,\cos\alpha ,0\right)=\left(-V\cos\alpha ,-V\cos\beta ,1\right).$$

According to (20), we obtain

(21) $$-V\cos\alpha u_{x}-V\cos\beta u_{y}+u_{t}=0.$$

Combining (18), (19) and (21), we find

(22) $$-V\cos\alpha {\left[u_{x}\right]}_{\left(x_{0},y_{0},t_{0}\right)}-V\cos\beta {\left[u_{y}\right]}_{\left(x_{0},y_{0},t_{0}\right)}+{\left[u_{t}\right]}_{\left(x_{0},y_{0},t_{0}\right)}=0,$$

and further

(23) $${\left[u_{t}\right]}_{\left(x_{0},y_{0},t_{0}\right)}=-q\left(x_{0},y_{0},t_{0}\right)V\cos^{2}\alpha -q\left(x_{0},y_{0},t_{0}\right)V\cos^{2}\beta =-q\left(x_{0},y_{0},t_{0}\right)V.$$

Combining (17) and (23), we get

(24) $${\left[u_{x^{\prime }x^{\prime }}\right]}_{\left(x_{0},y_{0},t_{0}\right)}=-q\left(x_{0},y_{0},t_{0}\right)V.$$

From (15), we have

(25) $${\left[u_{x^{\prime }y^{\prime }}\right]}_{\left(x_{0},y_{0},t_{0}\right)}={\left[u_{y^{\prime }x^{\prime }}\right]}_{\left(x_{0},y_{0},t_{0}\right)}=-q_{y^{\prime }}\left(x_{0},y_{0},t_{0}\right).$$

By the coordinate transformation (8), we obtain

(26) $$\left(\begin{array}{cc} u_{xx} & u_{xy}\\ u_{yx} & u_{yy} \end{array}\right)=\left(\begin{array}{cc} \cos\alpha & -\cos\beta \\ \cos\beta & \cos\alpha \end{array}\right)\left(\begin{array}{cc} u_{x^{\prime }x^{\prime }} & u_{x^{\prime }y^{\prime }}\\ u_{y^{\prime }x^{\prime }} & u_{y^{\prime }y^{\prime }} \end{array}\right)\left(\begin{array}{cc} \cos\alpha & \cos\beta \\ -\cos\beta & \cos\alpha \end{array}\right).$$

Combining (16), (24), (25), and (26), we obtain after simplification

(27) $$\left\{\begin{array}{c} {\left[u_{xx}\right]}_{\left(x_{0},y_{0},t_{0}\right)}=-q\left(x_{0},y_{0},t_{0}\right)V\cos^{2}\alpha +2q_{y^{\prime }}\left(x_{0},y_{0},t_{0}\right)\cos\alpha \cos\beta ,\\ {\left[u_{yy}\right]}_{\left(x_{0},y_{0},t_{0}\right)}=-q\left(x_{0},y_{0},t_{0}\right)V\cos^{2}\beta -2q_{y^{\prime }}\left(x_{0},y_{0},t_{0}\right)\cos\alpha \cos\beta . \end{array}\right.$$

For ease of concise description of the subsequent discretizations, we introduce the following jumps directly related to variables x,y,t. For $(x_{0},y_{0},t_{0})\in \Pi$,

(28) [u](x0,y0,t0)x=limε→0+(u(x0+ε,y0,t0)−u(x0−ε,y0,t0)),

(29) [u](x0,y0,t0)y=limε→0+(u(x0,y0+ε,t0)−u(x0,y0−ε,t0)),

(30) [u](x0,y0,t0)t=limε→0+(u(x0,y0,t0+ε)−u(x0,y0,t0−ε)).

It can be directly verified that

(31) [u](x0,y0,t0)x=sign(cosα)[u](x0,y0,t0),

(32) [u](x0,y0,t0)y=sign(cosβ)[u](x0,y0,t0),

(33) [u](x0,y0,t0)t=sign(V)[u](x0,y0,t0).

Define grid nodes in the domain Ω×[0,T] as

$$\left(x_{i},y_{j},t_{n}\right)=\left(a+ih_{x},c+jh_{y},n\tau \right),\;i=0,\ldots ,N_{x},\;j=0,\ldots ,N_{y},\;n=0,\ldots ,N_{t},$$

where $h_{x}=\left(b-a\right)/N_{x}$, $h_{y}=\left(d-c\right)/N_{y}$, and $\tau =T/N_{t}$ are the grid step sizes, and $N_{x},N_{y},N_{t}$ are the corresponding numbers of grid subdivisions. Denote $P_{i,j,n}=\left(x_{i},y_{j},t_{n}\right)$ and $u_{i,j}^{n}=u\left(x_{i},y_{j},t_{n}\right)$ for simplicity.

Taking grid node $\left(X_{i},Y_{j},t_{n}^{+}\right)\in \Omega \times \left(0,T\right]$ in Equation (4), for $i=1,\ldots ,N_{x}-1$, $j=1,\ldots ,N_{y}-1$, $n=1,\ldots ,N_{t}$, yields

(34) $$u_{t}\left(X_{i},Y_{j},t_{n}^{+}\right)=u_{xx}\left(X_{i},Y_{j},t_{n}^{+}\right)+u_{yy}\left(X_{i},Y_{j},t_{n}^{+}\right),$$

where $g(t^{+})$ denotes the right-hand limit of function g(t) at *t*, i.e., $g\left(t^{+}\right)=\lim_{\varepsilon \to 0^{+}}g\left(t+\varepsilon \right)$. If cosα>0, $X_{i}=x_{i}^{+}$, otherwise $X_{i}=x_{i}^{-}$. If cosβ>0, $Y_{j}=y_{j}^{+}$, otherwise $Y_{j}=y_{j}^{-}$. We use the symbols ${\left(u_{t}\right)}_{i,j,n}$, ${\left(u_{xx}\right)}_{i,j,n}$, and ${\left(u_{yy}\right)}_{i,j,n}$ to denote approximations of the three terms in (34), respectively, i.e.,

(35) $$u_{t}\left(X_{i},Y_{j},t_{n}^{+}\right)\approx {\left(u_{t}\right)}_{i,j,n},u_{xx}\left(X_{i},Y_{j},t_{n}^{+}\right)\approx {\left(u_{xx}\right)}_{i,j,n},u_{yy}\left(X_{i},Y_{j},t_{n}^{+}\right)\approx {\left(u_{yy}\right)}_{i,j,n}.$$

Replacing the terms in (34) with the approximations in (35) gives our finite difference discretization scheme:

(36) $${\left(u_{t}\right)}_{i,j,n}={\left(u_{xx}\right)}_{i,j,n}+{\left(u_{yy}\right)}_{i,j,n}.$$

**Remark 1.**  *Only within the discrete Equation (36), the symbol $u_{i,j}^{n}$ appearing in the expressions for ${\left(u_{t}\right)}_{i,j,n}$, ${\left(u_{xx}\right)}_{i,j,n}$ and ${\left(u_{yy}\right)}_{i,j,n}$ is understood as the numerical approximation to $u(x_{i},y_{j},t_{n})$.*

Next, we detail the process of determining the three approximations in the discretization scheme (36).

The approximation ${\left(u_{t}\right)}_{i,j,n}$ is determined according to the following two cases.

(i) If the line segment $P_{i,j,n-1}P_{i,j,n}$ does not intersect the plane Π, we set
  (37) $${\left(u_{t}\right)}_{i,j,n}=\frac{u_{i,j}^{n}-u_{i,j}^{n-1}}{\tau }.$$
  In this case, the second derivative of *u* with respect to *t* is bounded on $\left[t_{n-1},t_{n}\right]$, and the local truncation error of this discretization is O(τ).
(ii) If the line segment $P_{i,j,n-1}P_{i,j,n}$ intersects the plane Π at point $\left(x_{i},y_{j},\bar{t}\right)$, we construct the following auxiliary function:
  (38) u˜(xi,yj,t)=u(xi,yj,t)+(t−t¯)[ut](xi,yj,t¯)t,t<t¯,u(xi,yj,t),t¯≤t.
  It is easy to see that $\tilde{u}$ has a continuous first derivative with respect to *t*, its second derivative is bounded on $\left[t_{n-1},\bar{t}\right)\cup \left(\bar{t},t_{n}\right]$, and we have
  (39) $$u_{t}\left(x_{i},y_{j},t_{n}^{+}\right)={\tilde{u}}_{t}\left(x_{i},y_{j},t_{n}\right).$$
  Applying a finite difference discretization to ${\tilde{u}}_{t}\left(x_{i},y_{j},t_{n}\right)$, we obtain
  (40) u˜t(xi,yj,tn)=u˜(xi,yj,tn)−u˜(xi,yj,tn−1)τ+O(τ)=ui,jn−ui,jn−1τ−(tn−t¯)τ[ut](xi,yj,t¯)t+O(τ).
  We set
  (41) (ut)i,j,n=ui,jn−ui,jn−1τ+(tn−t¯)τ[ut]t(xi,yj,t¯),
  which yields a local truncation error of O(τ).

The approximation ${\left(u_{xx}\right)}_{i,j,n}$ is determined according to the following three cases.

(i) If the line segment $P_{i-1,j,n}P_{i+1,j,n}$ does not intersect the plane Π, we set
  (42) $$\begin{array}{c} {\left(u_{xx}\right)}_{i,j,n}=\frac{u_{i+1,j}^{n}-2u_{i,j}^{n}+u_{i-1,j}^{n}}{h_{x}^{2}}. \end{array}$$
  In this case, the third derivative of *u* with respect to *x* is bounded on $\left[x_{i-1},x_{i+1}\right]$, and the local truncation error is $O(h_{x}^{2})$.
(ii) If the line segment $P_{i-1,j,n}P_{i,j,n}$ intersects the plane Π at point $\left(\bar{x},y_{j},t_{n}\right)$ (when $\bar{x}=x_{i}$, an additional cosα<0 condition is required), we construct the following auxiliary function:
  (43) u^(x,yj,tn)=u(x,yj,tn)+(x−x¯)[ux](x¯,yj,tn)x+(x−x¯)22[uxx](x¯,yj,tn)x,x≤x¯,u(x,yj,tn),x¯<x.
  It is easy to see that $\hat{u}$ has a continuous second derivative with respect to *x*, its third derivative is bounded on $\left[x_{i-1},\bar{x}\right)\cup \left(\bar{x},x_{i+1}\right]$, and we have
  (44) $$\begin{array}{c} u_{xx}\left(x_{i},y_{j},t_{n}^{+}\right)={\hat{u}}_{xx}\left(x_{i},y_{j},t_{n}\right). \end{array}$$
  Applying a central difference scheme to discretize ${\hat{u}}_{xx}\left(x_{i},y_{j},t_{n}\right)$, we obtain
  (45) u^xx(xi,yj,tn)=u^(xi−1,yj,tn)−2u^(xi,yj,tn)+u^(xi+1,yj,tn)hx2+O(hx2)=ui+1,jn−2ui,jn+ui−1,jnhx2+(xi−1−x¯)[ux](x¯,yj,tn)xhx2+(xi−1−x¯)22hx2[uxx](x¯,yj,tn)x+O(hx2).
  We set
  (46) (uxx)i,j,n=ui+1,jn−2ui,jn+ui−1,jnhx2+(xi−1−x¯)[ux](x¯,yj,tn)xhx2+(xi−1−x¯)22hx2[uxx](x¯,yj,tn)x.
  This yields a local truncation error of $O(h_{x}^{2})$.
(iii) If the line segment $P_{i,j,n}P_{i+1,j,n}$ intersects the plane Π at point $\left(\bar{x},y_{j},t_{n}\right)$ (when $\bar{x}=x_{i}$, an additional cosα≥0 condition is required), we construct the following auxiliary function:
  (47) u^^(x,yj,tn)=u(x,yj,tn),x<x¯,u(x,yj,tn)−(x−x¯)[ux](x¯,yj,tn)x−(x−x¯)22[uxx](x¯,yj,tn)x,x¯≤x.
  It is easy to see that $\hat{\hat{u}}$ has a continuous second derivative with respect to *x*, its third derivative is bounded on $\left[x_{i-1},\bar{x}\right)\cup \left(\bar{x},x_{i+1}\right]$, and we have
  (48) $$u_{xx}\left(x_{i},y_{j},t_{n}^{+}\right)={\hat{\hat{u}}}_{xx}\left(x_{i},y_{j},t_{n}\right).$$
  Applying a central difference scheme to discretize ${\hat{\hat{u}}}_{xx}\left(x_{i},y_{j},t_{n}\right)$, we obtain
  (49) u^^xx(xi,yj,tn)=u^^(xi−1,yj,tn)−2u^^(xi,yj,tn)+u^^(xi+1,yj,tn)hx2+O(hx2)=ui+1,jn−2ui,jn+ui−1,jnhx2−(xi+1−x¯)[ux](x¯,yj,tn)xhx2−(xi+1−x¯)22hx2[uxx](x¯,yj,tn)x+O(hx2).
  We set
  (50) (uxx)i,j,n=ui+1,jn−2ui,jn+ui−1,jnhx2−(xi+1−x¯)[ux](x¯,yj,tn)xhx2−(xi+1−x¯)22hx2[uxx](x¯,yj,tn)x.
  This yields a local truncation error of $O(h_{x}^{2})$.

The determination process for the approximation ${\left(u_{yy}\right)}_{i,j,n}$ is similar to that for ${\left(u_{xx}\right)}_{i,j,n}$ and follows these three cases.

(i) If the line segment $P_{i,j-1,n}P_{i,j+1,n}$ does not intersect the plane Π, we set
  (51) $$\begin{array}{c} {\left(u_{yy}\right)}_{i,j,n}=\frac{u_{i,j+1}^{n}-2u_{i,j}^{n}+u_{i,j-1}^{n}}{h_{y}^{2}}. \end{array}$$
  The local truncation error is $O(h_{y}^{2})$.
(ii) If the line segment $P_{i,j-1,n}P_{i,j,n}$ intersects the plane Π at point $\left(x_{i},\bar{y},t_{n}\right)$ (when $\bar{y}=y_{j}$, an additional cosβ<0 condition is required), we set
  (52) (uyy)i,j,n=ui,j+1n−2ui,jn+ui,j−1nhy2+(yj−1−y¯)[uy](xi,y¯,tn)yhy2+(yj−1−y¯)22hy2[uyy](xi,y¯,tn)y.
  The local truncation error is $O(h_{y}^{2})$.
(iii) If the line segment $P_{i,j,n}P_{i,j+1,n}$ intersects the plane Π at point $\left(x_{i},\bar{y},t_{n}\right)$ (when $\bar{y}=y_{j}$, an additional cosβ≥0 condition is required), we set
  (53) (uyy)i,j,n=ui,j+1n−2ui,jn+ui,j−1nhy2−(yj+1−y¯)[uy](xi,y¯,tn)yhy2−(yj+1−y¯)22hy2[uyy](xi,y¯,tn)y.
  The local truncation error is $O(h_{y}^{2})$.

In summary, the scheme (36) constructed in this paper has different truncation errors in different regions. In the region adjacent to the heat source, the discretization has a truncation error of $O(\tau +h_{x}+h_{y})$, and the number of such discretizations is $O(N_{x}+N_{y})$. In the region far from the heat source, the discretization has a truncation error of $O(\tau +h_{x}^{2}+h_{y}^{2})$, and the number of such discretizations is $O(N_{x}N_{y})$. Therefore, the local first-order truncation errors does not dominate the overall convergence accuracy. Consequently, the final discrete scheme (36) achieves an overall error accuracy of $O(\tau +h_{x}^{2}+h_{y}^{2})$ globally.

Regarding the mesh ratio, we note that the implicit time discretization used in the scheme is unconditionally stable, hence there is no theoretical CFL-type restriction on $\tau /h^{2}$ for stability. However, to maintain the designed second-order global accuracy in practice, it is advisable to choose $\tau ∼O(h^{2})$ (where $h=\max\{h_{x},h_{y}\}$), so that the temporal and spatial errors remain balanced. All numerical experiments in Section 3 were performed with $\tau \leq 0.5h^{2}$.

## 3. Numerical Tests

This section validates the effectiveness and convergence accuracy of our proposed discretization scheme through a series of numerical experiments.

The simulation of the moving line source reflects the temperature evolution under concentrated heat input along a moving path, as seen in processes such as welding or laser cladding. The results for the moving plane source can inform thermal management in additive manufacturing or laser surface treatment, where heat deposition over a moving area is critical for microstructure control and residual stress prediction. Moreover, accurately capturing the temperature gradient and heat flux jump near the source is essential for predicting phase transformation, thermal distortion, and local entropy production in such processes.

### 3.1. Two-Dimensional Problems

Consider problem (4) with Ω=[0,2]×[0,2], T=1, and $N_{x}=N_{y}=N$. Let $u^{N}$ be the numerical solution with grid number *N*, and $u^{2N}$ be the numerical solution obtained after doubling the grid resolution. The errors in the discrete $L^{1}$ and $L^{\infty }$ norms, and the convergence order in the discrete $L^{1}$ norm, are defined as follows:

(54) $$Err_{1}^{N}=\left|\right|u^{N}-u^{2N}{\left|\right|}_{1}=h_{x}h_{y}\sum_{i=1}^{N_{x}}\sum_{j=1}^{N_{y}}\left|u_{i,j}^{N}-u_{2i-1,2j-1}^{2N}\right|,$$

(55) $$Err_{\infty }^{N}=\left|\right|u^{N}-u^{2N}{\left|\right|}_{\infty }=\max_{\begin{array}{c} 1\leq i\leq N_{x}\\ 1\leq j\leq N_{y} \end{array}}\left|u_{i,j}^{N}-u_{2i-1,2j-1}^{2N}\right|.$$

and

(56) $$Ord_{1}^{N}=\log_{2}\;\left(\frac{Err_{1}^{N}}{Err_{1}^{2N}}\right).$$

**Example 1.**  *The moving heat source is given by*

$$L_{t}=\left\{\left(x,y\right);\sin\left(\frac{\pi }{5}\right)x-\cos\left(\frac{\pi }{5}\right)y+t=0\right\}.$$

**Figure 2:**
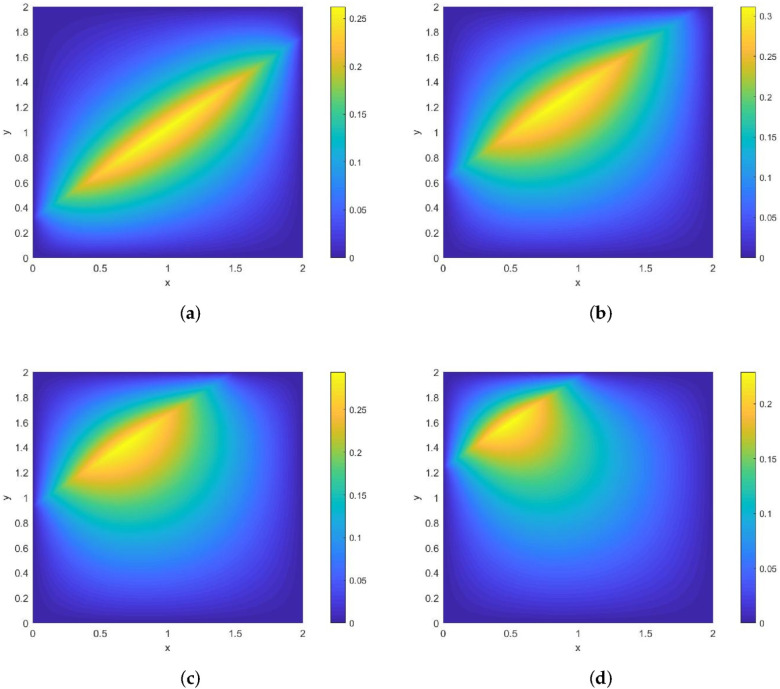
Contour plots of the numerical solution at different times for q=1, where (**a**) corresponds to the contour plot at t=0.25, (**b**) to t=0.5, (**c**) to t=0.75, and (**d**) to t=1.

**Figure 3:**
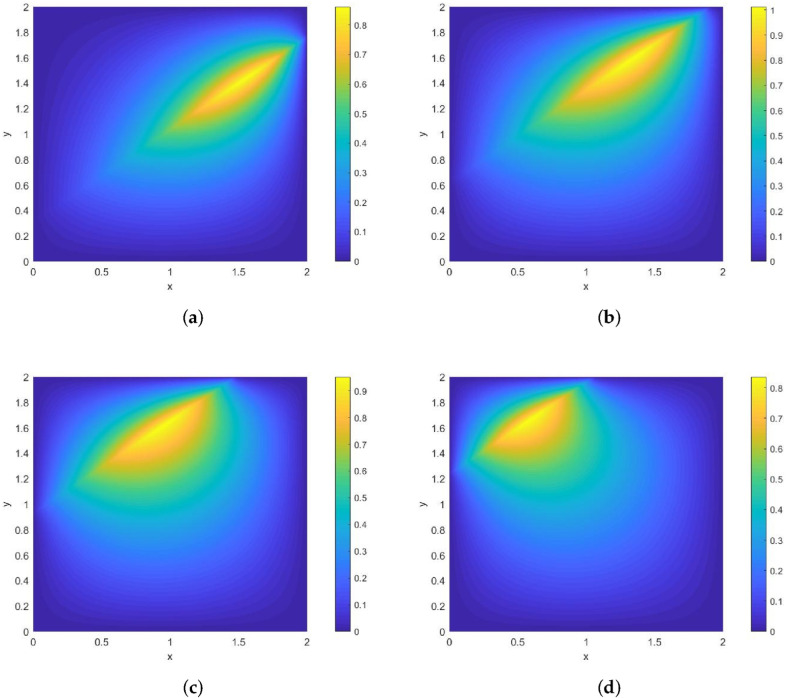
Contour plots of the numerical solution at different times for $q=x^{2}+y^{2}+t^{2}$, where (**a**) corresponds to t=0.25, (**b**) to t=0.5, (**c**) to t=0.75, and (**d**) to t=1.

*Its direction of motion is $\left(-\sin\frac{\pi }{5},\cos\frac{\pi }{5}\right)$, with a speed of 1. Figure 2 and Figure 3 show contour plots of the numerical solution at different times for heat source intensities q=1 and $q=x^{2}+y^{2}+t^{2}$, respectively, obtained using a grid with N=256, $N_{t}=32,786$. The corresponding errors and convergence orders are presented in Table 1.*

**Example 2.**  *The moving heat source is given by*

$$L_{t}=\left\{\left(x,y\right);-x+0.5t+0.5=0\right\}.$$

**Figure 4:**
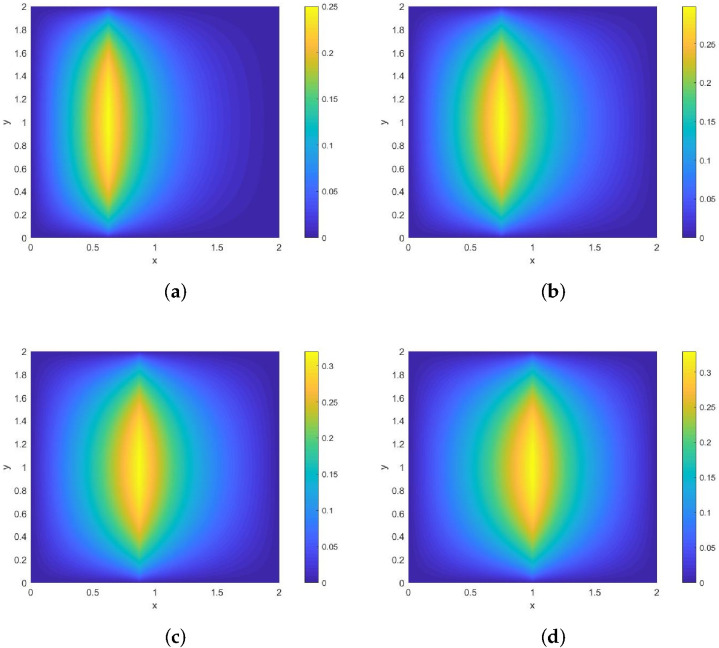
Contour plots of the numerical solution at different times for q=1, where (**a**) corresponds to t=0.25, (**b**) to t=0.5, (**c**) to t=0.75, and (**d**) to t=1.

**Figure 5:**
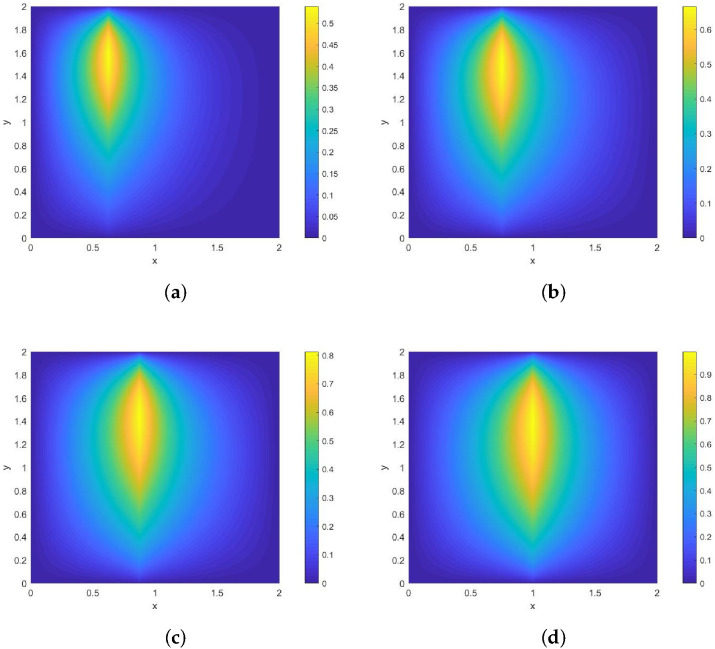
Contour plots of the numerical solution at different times for $q=x^{2}+y^{2}+t^{2}$, where (**a**) corresponds to t=0.25, (**b**) to t=0.5, (**c**) to t=0.75, and (**d**) to t=1.

*Its direction of motion is (1,0), with a speed of 1. Figure 4 and Figure 5 show contour plots of the numerical solution at different times for heat source intensities q=1 and $q=x^{2}+y^{2}+t^{2}$, respectively, obtained using a grid with N=256, $N_{t}=32,786$. The corresponding errors and convergence orders are presented in Table 2.*

**Example 3.**  *The moving heat source is given by*

$$L_{t}=\left\{\left(x,y\right);x=0.8-\frac{\sqrt{2}}{2}t+\frac{\sqrt{2}}{2}s,y=0.2+\frac{\sqrt{2}}{2}t+\frac{\sqrt{2}}{2}s,s\in \left[0,1\right]\right\}.$$

**Figure 6:**
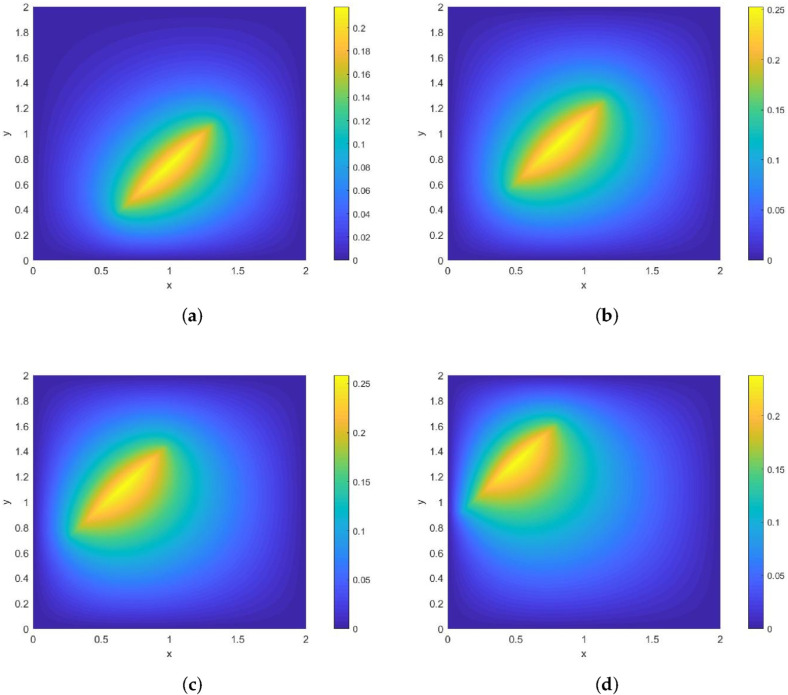
Contour plots of the numerical solution at different times for q(s)=1, where (**a**) corresponds to t=0.25, (**b**) to t=0.5, (**c**) to t=0.75, and (**d**) to t=1.

**Figure 7:**
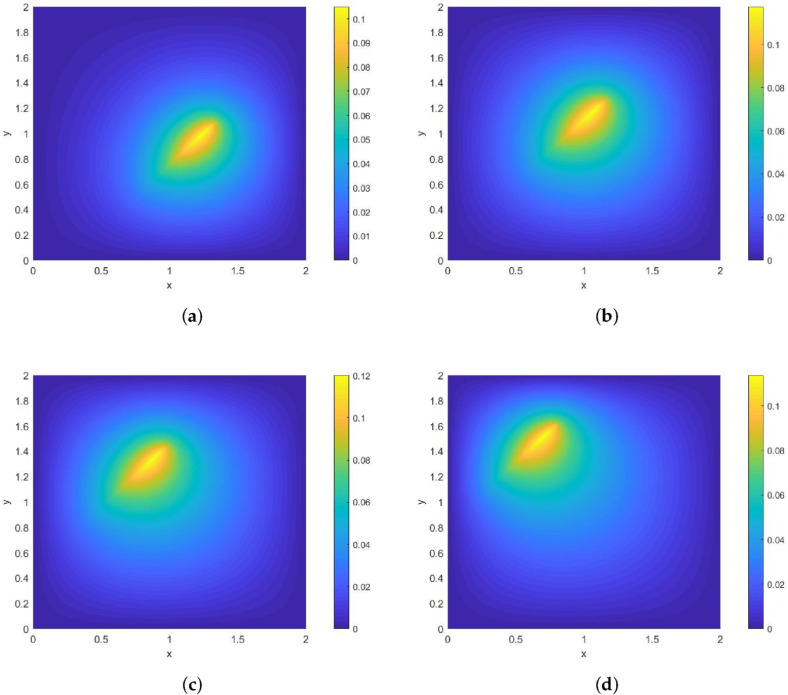
Contour plots of the numerical solution at different times for $q\left(s\right)=s^{2}$, where (**a**) corresponds to t=0.25, (**b**) to t=0.5, (**c**) to t=0.75, and (**d**) to t=1.

*Its direction of motion is $\left(-\frac{\sqrt{2}}{2},\frac{\sqrt{2}}{2}\right)$, with a speed of 1. Figure 6 and Figure 7 show contour plots of the numerical solution at different times for heat source intensities q(s)=1 and $q\left(s\right)=s^{2}$, respectively, obtained using a grid with N=256, $N_{t}=32,786$. The corresponding errors and convergence orders are presented in Table 3.*

### 3.2. Three-Dimensional Problems

Consider the following initial-boundary value problem with a moving plane heat source:

(57) $$\left\{\begin{array}{ccc} u_{t}-\Delta u=\delta_{L_{t}}\left(x,y,z\right)q\left(x,y,z,t\right), & \; & \left(x,y,z\right)\in \Omega ={\left[0,2\right]}^{3},\;0<t\leq 1,\\ u(x,y,z,0)=0, & \; & (x,y,z)\in \Omega ,\\ {u|}_{\partial \Omega }=0. & \; \end{array}\right.$$

The three-dimensional discretization scheme can be directly derived from the two-dimensional scheme, the details of which we omit here. We present the numerical results directly. The error norms for the three-dimensional problem are defined as

(58) $$Err_{1}^{N}=\left|\right|u^{N}-u^{2N}{\left|\right|}_{1}=h_{x}h_{y}h_{z}\sum_{i=1}^{N_{x}}\sum_{j=1}^{N_{y}}\sum_{k=1}^{N_{z}}\left|u_{i,j,k}^{N}-u_{2i-1,2j-1,2k-1}^{2N}\right|,$$

(59) $$Err_{\infty }^{N}={\left|u^{N}-u^{2N}\right|}_{\infty }=\max_{\begin{array}{c} 1\leq i\leq N_{x}\\ 1\leq j\leq N_{y}\\ 1\leq k\leq N_{z} \end{array}}\left|u_{i,j,k}^{N}-u_{2i-1,2j-1,2k-1}^{2N}\right|.$$

**Example 4.**  *The moving heat source is given by*

$$L_{t}=\left\{\left(x,y,z\right);x+y+z+t-4=0\right\}.$$

**Figure 8:**
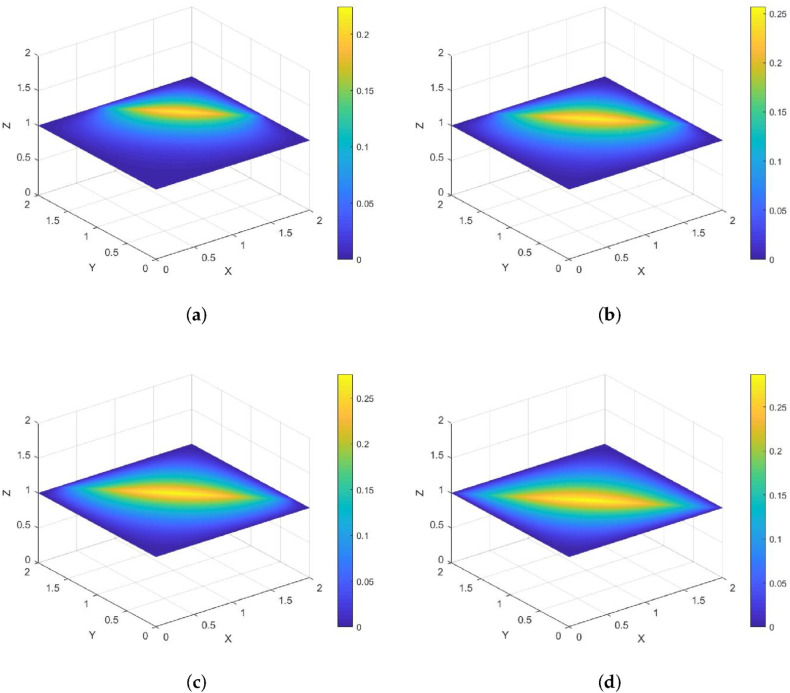
Contour plots of the numerical solution on the cross-section z=1 at different times for q=1, where (**a**) corresponds to t=0.25, (**b**) to t=0.5, (**c**) to t=0.75, and (**d**) to t=1.

**Figure 9:**
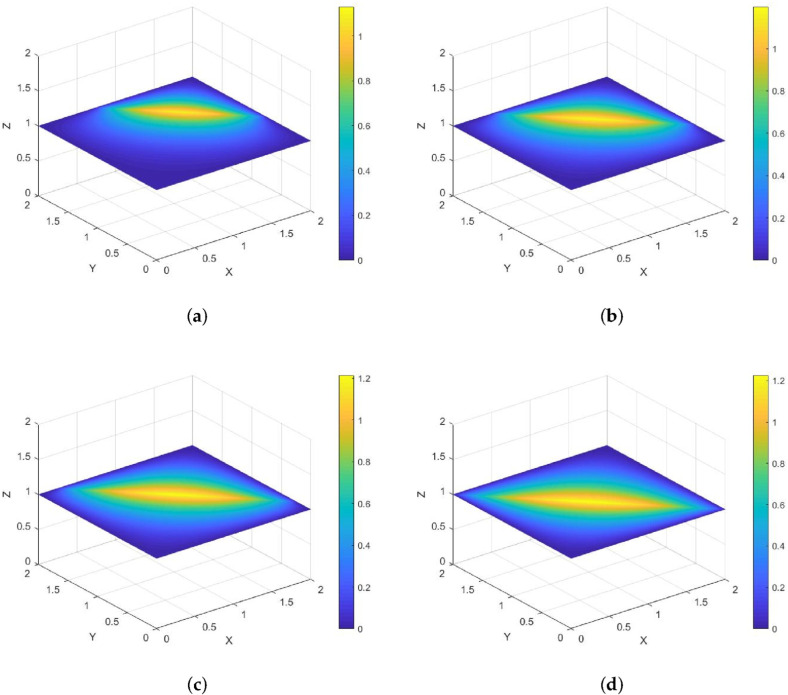
Contour plots of the numerical solution on the cross-section z=1 at different times for $q=x^{2}+y^{2}+z^{2}+t^{2}$, where (**a**) corresponds to t=0.25, (**b**) to t=0.5, (**c**) to t=0.75, and (**d**) to t=1.

*Its direction of motion is $\left(-\frac{\sqrt{3}}{3},-\frac{\sqrt{3}}{3},-\frac{\sqrt{3}}{3}\right)$, with a speed of $\frac{\sqrt{3}}{3}$. Figure 8 and Figure 9 show contour plots of the numerical solution on the cross-section z=1 at different times for heat source intensities q=1 and $q=x^{2}+y^{2}+z^{2}+t^{2}$, respectively, obtained using a grid with N=128, $N_{t}=8192$. The corresponding errors and convergence orders are presented in Table 4.*

**Example 5.**  *The moving heat source is given by*

$$L_{t}=\left\{\left(x,y,z\right);x=1.5-t,{\left(y-0.5\right)}^{2}+{\left(z-0.5\right)}^{2}\leq 0.5^{2}\right\}.$$

**Figure 10:**
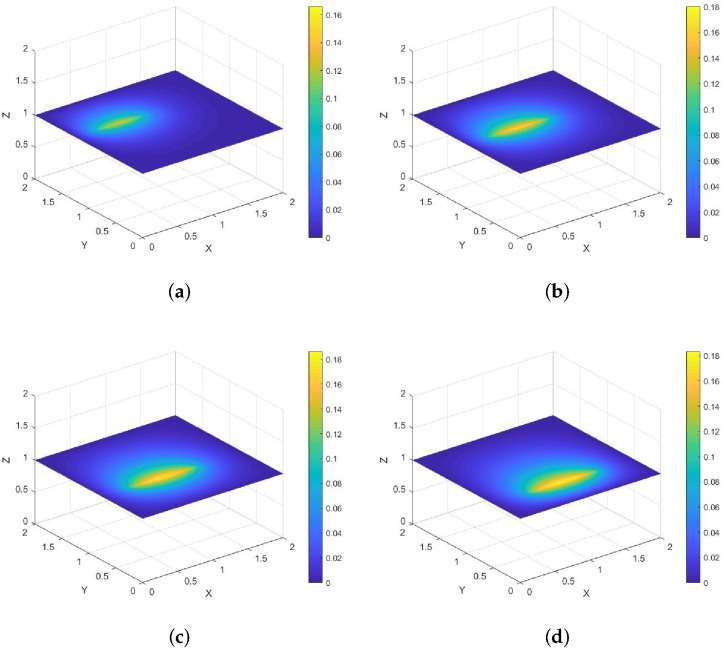
Contour plots of the numerical solution on the cross-section z=1 at different times for q=1, where (**a**) corresponds to t=0.25, (**b**) to t=0.5, (**c**) to t=0.75, and (**d**) to t=1.

**Figure 11:**
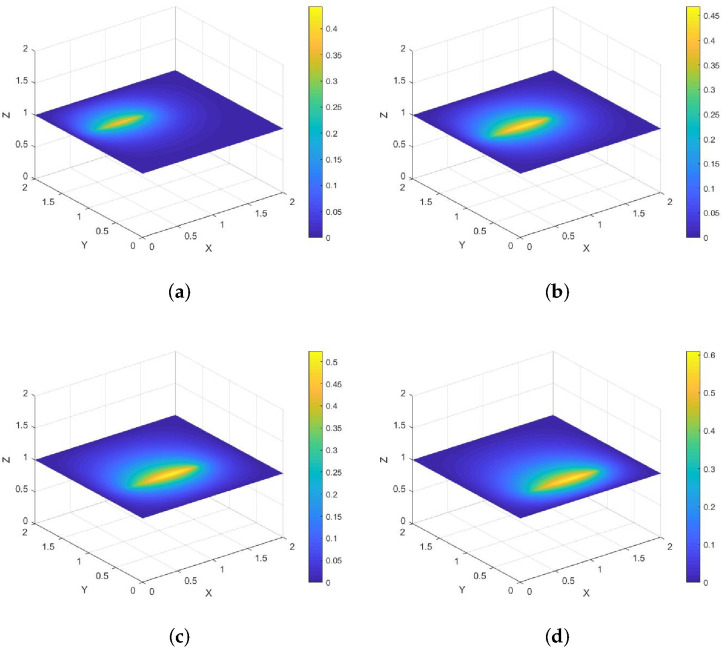
Contour plots of the numerical solution on the cross-section z=1 at different times for $q=x^{2}+y^{2}+z^{2}+t^{2}$, where (**a**) corresponds to t=0.25, (**b**) to t=0.5, (**c**) to t=0.75, and (**d**) to t=1.

*Its direction of motion is (−1,0,0), with a speed of 1. Figure 10 and Figure 11 show contour plots of the numerical solution on the cross-section z=1 at different times for heat source intensities q=1 and $q=x^{2}+y^{2}+z^{2}+t^{2}$, respectively, obtained using a grid with N=128, $N_{t}=8192$. The corresponding errors and convergence orders are presented in Table 5.*

## 4. Conclusions

This paper presents a partitioned finite difference method for transient heat conduction with moving line and plane heat sources. The method analytically handles the solution singularity using jump conditions, applying first-order discretization near the source and standard second-order schemes elsewhere. This hybrid strategy ensures second-order spatial convergence overall, as confirmed by numerical tests on straight lines, segments, planes, and circular surfaces. The method provides an efficient and accurate tool for simulating heat diffusion from moving singularities, useful for studying energy distribution in related non-equilibrium processes. Its sparse discretization also supports future extensions to problems with convection, nonlinearities, or multiphysics coupling.

## Figures and Tables

**Table 1:** Errors and convergence orders.

*q*	1			$x^{2}+y^{2}+t^{2}$		
N	${\mathrm{Err}}_{1}^{N}$	${\mathrm{Err}}_{\infty }^{N}$	${\mathrm{Ord}}_{1}^{N}$	${\mathrm{Err}}_{1}^{N}$	${\mathrm{Err}}_{\infty }^{N}$	${\mathrm{Ord}}_{1}^{N}$
16	7.73 $\times \;10^{-3}$	1.29 $\times \;10^{-2}$	—	2.50 $\times \;10^{-2}$	5.54 $\times \;10^{-2}$	—
32	1.80 $\times \;10^{-3}$	3.68 $\times \;10^{-3}$	2.09	5.69 $\times \;10^{-3}$	1.26 $\times \;10^{-2}$	2.13
64	4.60 $\times \;10^{-4}$	9.52 $\times \;10^{-4}$	1.97	1.50 $\times \;10^{-3}$	5.72 $\times \;10^{-3}$	1.91
128	1.15 $\times \;10^{-4}$	1.92 $\times \;10^{-4}$	1.99	3.76 $\times \;10^{-4}$	1.17 $\times \;10^{-3}$	2.00
256	2.97 $\times \;10^{-5}$	3.78 $\times \;10^{-4}$	1.95	9.52 $\times \;10^{-5}$	9.56 $\times \;10^{-4}$	1.98

**Table 2:** Errors and convergence orders.

*q*	1			$x^{2}+y^{2}+t^{2}$		
N	${\mathrm{Err}}_{1}^{N}$	${\mathrm{Err}}_{\infty }^{N}$	${\mathrm{Ord}}_{1}^{N}$	${\mathrm{Err}}_{1}^{N}$	${\mathrm{Err}}_{\infty }^{N}$	${\mathrm{Ord}}_{1}^{N}$
16	9.35 $\times \;10^{-3}$	1.34 $\times \;10^{-2}$	—	2.95 $\times \;10^{-2}$	7.20 $\times \;10^{-2}$	—
32	1.82 $\times \;10^{-3}$	5.62 $\times \;10^{-3}$	2.35	6.01 $\times \;10^{-3}$	3.27 $\times \;10^{-2}$	2.29
64	3.80 $\times \;10^{-4}$	2.59 $\times \;10^{-3}$	2.26	1.26 $\times \;10^{-3}$	1.54 $\times \;10^{-2}$	2.24
128	8.37 $\times \;10^{-5}$	1.24 $\times \;10^{-3}$	2.18	2.79 $\times \;10^{-4}$	7.47 $\times \;10^{-3}$	2.18
256	1.84 $\times \;10^{-5}$	6.14 $\times \;10^{-4}$	2.17	5.80 $\times \;10^{-5}$	3.68 $\times \;10^{-3}$	2.26

**Table 3:** Errors and convergence orders.

q(s)	1			$s^{2}$		
N	${\mathrm{Err}}_{1}^{N}$	${\mathrm{Err}}_{\infty }^{N}$	${\mathrm{Ord}}_{1}^{N}$	${\mathrm{Err}}_{1}^{N}$	${\mathrm{Err}}_{\infty }^{N}$	${\mathrm{Ord}}_{1}^{N}$
16	5.43 $\times \;10^{-3}$	1.30 $\times \;10^{-2}$	—	4.68 $\times \;10^{-3}$	1.62 $\times \;10^{-2}$	—
32	2.48 $\times \;10^{-3}$	6.04 $\times \;10^{-3}$	1.13	1.73 $\times \;10^{-3}$	5.40 $\times \;10^{-3}$	1.43
64	4.92 $\times \;10^{-4}$	6.70 $\times \;10^{-3}$	2.33	3.33 $\times \;10^{-4}$	6.28 $\times \;10^{-3}$	2.37
128	1.24 $\times \;10^{-4}$	3.82 $\times \;10^{-3}$	1.97	8.75 $\times \;10^{-5}$	3.76 $\times \;10^{-3}$	1.92
256	3.76 $\times \;10^{-5}$	2.32 $\times \;10^{-3}$	1.73	2.65 $\times \;10^{-5}$	2.35 $\times \;10^{-3}$	1.72

**Table 4:** Errors and convergence orders.

*q*	1			$x^{2}+y^{2}+z^{2}+t^{2}$		
N	${\mathrm{Err}}_{1}^{N}$	${\mathrm{Err}}_{\infty }^{N}$	${\mathrm{Ord}}_{1}^{N}$	${\mathrm{Err}}_{1}^{N}$	${\mathrm{Err}}_{\infty }^{N}$	${\mathrm{Ord}}_{1}^{N}$
16	5.46 $\times \;10^{-3}$	4.69 $\times \;10^{-3}$	—	2.94 $\times \;10^{-2}$	2.55 $\times \;10^{-2}$	—
32	1.56 $\times \;10^{-3}$	2.42 $\times \;10^{-3}$	1.80	8.44 $\times \;10^{-3}$	1.36 $\times \;10^{-2}$	1.80
64	4.11 $\times \;10^{-4}$	1.26 $\times \;10^{-3}$	1.92	2.22 $\times \;10^{-3}$	7.31 $\times \;10^{-3}$	1.92
128	1.05 $\times \;10^{-4}$	6.52 $\times \;10^{-4}$	1.97	5.69 $\times \;10^{-4}$	3.83 $\times \;10^{-3}$	1.96

**Table 5:** Errors and convergence orders.

*q*	1			$x^{2}+y^{2}+z^{2}+t^{2}$		
N	${\mathrm{Err}}_{1}^{N}$	${\mathrm{Err}}_{\infty }^{N}$	${\mathrm{Ord}}_{1}^{N}$	${\mathrm{Err}}_{1}^{N}$	${\mathrm{Err}}_{\infty }^{N}$	${\mathrm{Ord}}_{1}^{N}$
16	8.84 $\times \;10^{-3}$	1.52 $\times \;10^{-2}$	—	3.37 $\times \;10^{-2}$	6.48 $\times \;10^{-2}$	—
32	1.73 $\times \;10^{-3}$	8.28 $\times \;10^{-3}$	2.35	6.33 $\times \;10^{-3}$	3.64 $\times \;10^{-2}$	2.41
64	7.42 $\times \;10^{-4}$	4.86 $\times \;10^{-3}$	1.22	2.46 $\times \;10^{-3}$	2.20 $\times \;10^{-2}$	1.35
128	9.79 $\times \;10^{-5}$	2.82 $\times \;10^{-3}$	2.92	3.37 $\times \;10^{-4}$	1.26 $\times \;10^{-2}$	2.87

## Data Availability

The algorithm implementation code and related data supporting the findings of this study are available in a CSDN repository at: https://download.csdn.net/download/weixin_51130032/92570878, accessed on 14 December 2025.
